# Editorial: Multiscale Approach to Assess Forest Vulnerability

**DOI:** 10.3389/fpls.2020.00744

**Published:** 2020-06-05

**Authors:** Giovanna Battipaglia, Andreas Rigling, Veronica De Micco

**Affiliations:** ^1^Department of Environmental, Biological and Pharmaceutical Sciences and Technologies University of Campania L. Vanvitelli, Caserta, Italy; ^2^Swiss Federal Research Institute WSL, Birmensdorf, Switzerland; ^3^Institute of Terrestrial Ecosystems, ETH Zurich, Zurich, Switzerland; ^4^Department of Agricultural Sciences, University of Naples Federico II, Naples, Italy

**Keywords:** climate change, forest vulnerability, forest dynamics, forest growth, drought mortality

In recent decades, forest, vulnerability to climate change is rapidly increasing worldwide; forest dieback episodes have been recorded in all biomes (Allen et al., 2010; Adams et al., 2017). In forest ecosystems a high probability of intensified occurrence of extreme events, such as heat waves, droughts, fires, flooding or pest outbreaks is expected (Seidl et al., 2011; FAO, 2018).

Forest vulnerability can be defined as the degree to which a forest ecosystem is susceptible to adverse effects of climate change. Thus, vulnerability is a function of the climate variation which the forest is exposed to (exposure), its sensitivity, and its adaptive capacity to respond to the potential impacts (Figure 1A).

**Figure 1:**
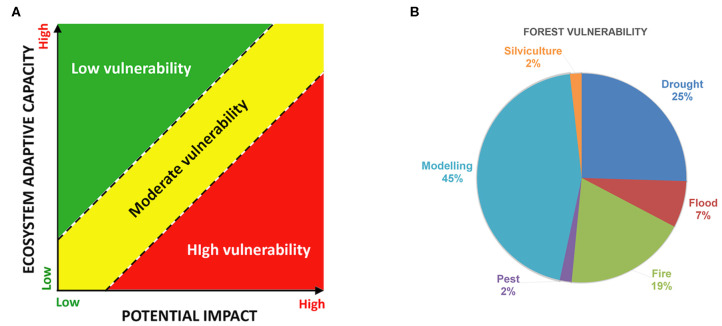
**(A)** Vulnerability is a combination of adaptative capacity and potential impact [modified by Allen et al. (2010)]; **(B)** Percentage of papers published from 1999 to 2019 dealing with forest vulnerability to different environmental stressors, as indexed in the Web of Science™ database (Thomson Reuters).

Several cases of widespread dieback and increased mortality rates have been described for different tree and shrub species, revealing the high vulnerability of some forest ecosystems, manifested as a loss in tree vigor, growth decline and sometimes tree death (Montoro Girona et al., 2019). The increasing forest vulnerability and occurrence of dieback cases, extended to a larger scale, can have the potential to rapidly alter forest functioning and respective ecosystem services, with important implications on the carbon-water balance, plant community composition, and tree population dynamics (Rewald et al., 2020). Thus, assessing increased forest vulnerability is a challenging issue and this Research Topic (RT) aimed to contribute to close research gaps in identifying vulnerable species and ecosystems, understanding factors triggering forest vulnerability, estimating the adaptive capacity of forest ecosystems to climate change, and identifying possible effects of forestry practices on forest vulnerability.

The 17 articles collected in this RT underlined how the assessment of vulnerability is a complex matter, leading to a common agreement on the key role of interdisciplinary research and networking to fasten the understanding of the phenomena and related factors affecting tree mortality as well as to develop sustainable forest management practices under climate change.

The majority of papers deal with forest vulnerability to extreme drought. Different species- and provenance specific-impacts, site conditions and stand compositions are considered as factors

affectingf plant growth and forest mortality. Wang et al. investigated the vulnerability of *Quilian junipers*, a widespread species of the Northeastern Tibetan Plateau, showing negative growth trends when facing extreme drought events. The authors underlined that the oldest populations are the most vulnerable, characterized by the lowest resistance values, the narrowest annual rings, and the highest proportion of missing rings during the drought years.

The importance o understanding and selecting the more resilient provenances of Scots pine to alleviate pressures of climate change on forest ecosystems, was investigated by Seidel et al. The authors demonstrated that southern provenances of Scots pine were better adapted to drought conditions than northern ones, showing a less severe drought response and exhibiting morphological characteristics associated with drought resistance. Klisz et al. studied provenance-specific climate sensitivity of Norway spruce growing in the natural range and at the climatic margin of species distribution. Results showed that the marginality of site has to be considered when evaluating climate sensitivity, since the provenance growing at the margin of distribution was much more affected by drought than the one growing in the center of the distribution.

Not only species, but also site conditions and stand structure, influenced the responses to drought of pure and mixed forests of Mediterranean forests. Indeed, Zalloni et al. demonstrated that the impact of drought on *Quercus ilex* growing in a pure or mixed stand with *Pinus pinea*, in Southern Italy, is strongly related to the characteristics of the stands. In a mixed stand, where *Q. ilex* trees are young and stand density is high, thinning of *P. pinea*, is advisable to limit inter-specific competition for resources and to promote *Q. ilex* wood growth. On the contrary, when stand density is lower, promoting the co-existence of *Q. ilex* and *P. pinea* could facilitate complementarity in resource use. In the Mediterranean forest, considered particularly vulnerable to climate change (FAO, 2018), Gazol et al. showed a potential compositional shift in two large Spanish forests as a consequence of recent warming trends and the severe droughts, including that occurring in 2012, which caused die-off in both forests. The authors findings warned against the local extinction of some tree populations near their southernmost distribution limits as in the *P. sylvestris* forest studied which may become more vulnerable to forecasted aridification trends.

Moving to tropical ecosystems, a large dendrometer dataset was analyzed (Raffelsbauer et al.) to understand growth recovery after dry events in Ecuador. Even if the precipitations are abundant, dry spells occur regularly during so-called “Veranillo del Niño” (VdN) periods in October-November. Analyzing two broadleaved species, the authors showed that higher frequency of drought might increase inter-species competition and species-specific mortality and might finally alter the species composition of the ecosystem. A second study carried out in the tropics was based on wood traits (Islam et al.) and covered different tree species. The authors demonstrated different adjustment strategies among the species, probably due to the differences in evolutionary trends in xylem traits.

The importance of large databases in assessing general growth patterns of species and mortality rate is highlighted in the studies of Etzold et al. and Cailleret et al. Indeed, Etzold et al. studying 276 permanent plots across Switzerland, assessed mortality rates of five dominant species throughout the last century (1898–2013) and examined factors driving mortality change. Surprisingly the authors found that mortality rates increased only slightly over the last ~120 years, which could be mainly related to changes in stand structure. This suggests that Swiss forests have been resilient to recent climate change so far and that changes in species composition might occur more gradually. A large tree-ring database was used by Cailleret et al. to explore forest mortality and to provide a robust method for estimating early-warning signals of tree mortality. The authors reported a decrease in growth rates, an increase in inter-annual growth variability and a decrease in growth synchrony in gymnosperm species, that could be used as powerful predictors of mortality risk, but not necessarily so for angiosperms. Their results are in agreement with several studies where species distributional range and forest composition changes are predicted (Sykes and Prentice, 1996; Rigling et al., 2013; Fekete et al., 2017; Scherrer et al., 2017).

Buras and Menzel projected tree species composition changes, according to future climate scenarios. Their results indicate significant changes in European forest species composition: species richness decline in the Mediterranean and Central European lowlands, while increasing diversity in Scandinavian and Central European high-elevation forests.

The impact of climate change on the ecosystem services (ES) of boreal forests are evaluated by Holmberg et al., applying two dynamic ecosystem models. Even if they found both, beneficial and detrimental consequences, the main output of their paper was the high range of uncertainty in future provision of boreal forest ES.

Apart from climate impacts, especially drought, other important factors, including fire, flood or pest outbreaks, can trigger forest vulnerability, but their impact is less studied. According to the Web of Science™ database (Thomson Reuters) (Figure 1B), considering the last 20 years (1999–2019) the majority of studies are related to drought and models, while only a small percentage is related to the other non-climatic factors probably because of the difficulty to disentangle the effects of climate and biotic and abiotic factors on forest dieback (Niccoli et al.). A part from models, this percent distribution of papers among subjects is reflected in this RT as well, with only one study dealing with the effects of fire on forest growth, (Niccoli et al.) one paper on the effects of floodplain on forest productivity, (Netsvetov et al.) two studies analyzing the relationships between pest outbreaks and tree mortality (Navarro et al.; Rossi et al.) and two contributions reporting on the effects of silvicultural practices in reducing forest vulnerability (Girona et al.; Thyroff et al.). All those papers presented novel and interesting results in their topics. Indeed, Niccoli et al. used a multidisciplinary approach to clarify the ecophysiological processes allowing *Pinus pinaster* to survive after a severe wildfire, while Netsvetov et al. demonstrated that trees growing in areas exposed to urban development are the most susceptible to downside effects of river regulation. The paper of Navarro et al. was the first evidence of a northward shift of the distribution area of spruce budworm, as well as the first spatio-temporal reconstruction at the landscape level in Quebec, highlighting the complexity of outbreak dynamics and, at the same time, the necessity of this approach for assessing boreal forest adaptation and modification to future climate change. Rossi et al. studied how the spruce budworm outbreak affected the responses of *Picea mariana* to a subsequent thinning. Their dendrochronological analyses underlined that the effect of thinning on tree-ring width of *Picea mariana* was independent of the growth reduction that trees had experienced during the outbreak.

To cover the importance of new silvicultural practices in reducing forest vulnerability, Girona et al. presented a regeneration study in *Picea mariana* stands. Their findings confirmed, for the first time, that experimental shelterwood and seed-tree harvesting followed by scarification allow the establishment of an abundant black spruce regeneration in North American boreal forests and can be a viable silvicultural alternative to clear-cutting. Finally, the study of Thyroff et al. demonstrated the high plasticity of *Quercus virginiana* to resource availability (such as light) and the importance of applying more than one silvicultural treatment to reduce the need for costly competing vegetation control.

Taken together, the 17 contributions to this RT cover many aspects of timely research on forest vulnerability to different environmental stressors. Notwithstanding the numerous claims on the need to apply multidisciplinary and multi-scale approaches to evaluate forest ecosystem condition, to assess vulnerability to environmental stress and adopt countermeasures against mortality, there is still a lack of integration among disciplines, data fusion capacities and of collaborative actions at regional and global scales (Battipaglia et al., 2014; De Micco et al., 2019).

Therefore, as an output of this RT, many outstanding research gaps were evidenced which highlighted the following issues:

- systematic and holistic research approaches covering key aspects including more interdisciplinary studies are needed;
- current research, more focused on causes and mechanisms related to forest vulnerability, should be more directed toward forest management issues to improve ecosystems resilience and resistance;
- there is a need for better understanding and predicting tree mortality, growth and recruitment in response to climate variation, which is essential to improve vegetation and carbon cycle models, also establishing long-term forest monitoring networks and studying the large-scale effects in a spatially explicit manner;
- forest vulnerability to environmental stressors often derives from local studies or spatially and temporally coarse datasets that do not allow a comprehensive ecological analysis across administrative boundaries;
- there is a need of better integration among direct observations, field data and modeling approach to gain concrete information for forest management;
- a consistent understanding of climate change impacts on tree and population dynamics remains elusive due to the difficulty of obtaining data from systematic field studies at different scales from local to regional importance of continuous tree monitoring to get instant information on tree functioning;
- studies on belowground biomass activity (belowground C allocation) in function of changes in temperature, water availability as well as management practices are lacking;
- there is a need for understanding the role of single and combined adaptive traits in growth efficiency and plasticity;
- there is a need for whole-plant approaches linking above- and below-ground growth dynamics in the continuum soil-plant-atmosphere.

To fill these gaps, making synergies among disciplines to speed-up the process of knowledge achievement, a networking effort is needed as also highlighted recently in other contexts. Networking also across disciplines and communities would be highly welcome to achieve an in-depth understanding of forest functioning through the whole soil-plant-atmosphere pathway allowing for predictions on forest destiny and ES under various climate change scenarios.

## Author Contributions

GB, AR, and VD: conceptualization. GB: writing original draft. All authors: writing—review & editing.

## Conflict of Interest

The authors declare that the research was conducted in the absence of any commercial or financial relationships that could be construed as a potential conflict of interest.
